# Mechanism of Benzene Hydroxylation on Tri-Iron Oxo-Centered Cluster-Based Metal–Organic Frameworks

**DOI:** 10.1021/acs.jpcc.3c06423

**Published:** 2023-11-24

**Authors:** Jenny G. Vitillo, Madhuresh Choudhary, Matthew C. Simons, Laura Gagliardi, Aditya Bhan

**Affiliations:** † Department of Science and High Technology and INSTM, Università Degli Studi Dell’Insubria, Via Valleggio 9, Como I-22100, Italy; ‡ Department of Chemical Engineering and Materials Science, University of Minnesota, 421 Washington Avenue S.E., Minneapolis, Minnesota 55455, United States; § Department of Chemistry, Pritzker School of Molecular Engineering, James Franck Institute, University of Chicago, Chicago, Illinois 60637, United States

## Abstract

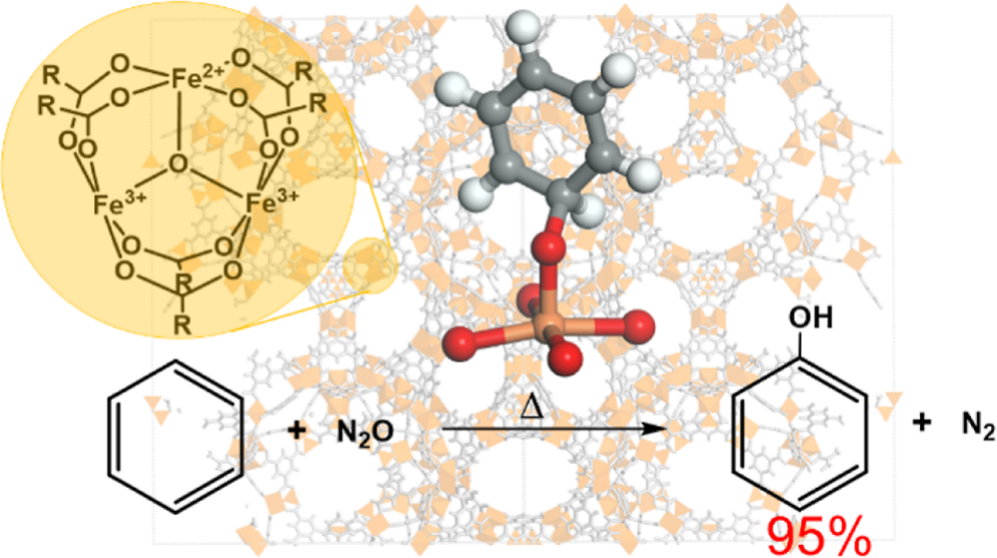

High-valent Fe(IV)-oxo species derived upon reactions of N_2_O with Fe(II) centers—embedded in the framework of tri-iron oxo-centered-based metal–organic frameworks (MOFs)— selectively affect the conversion of benzene-to-phenol via electrophilic addition to arene C–H bonds akin to oxygen transfer mechanisms in the P450 enzyme. The Fe(II) species identified by Mössbauer spectroscopy can be titrated in situ by the addition of NO to completely suppress benzene oxidation, verifying the relevance of Fe(II) centers. Observed inverse kinetic isotope effects in benzene hydroxylation preclude the involvement of H atom transfer steps from benzene to the Fe(IV)-oxo species and instead suggest that the electrophilic iron-oxo group adds to an sp^2^ carbon of benzene, resulting in a change in the hybridization from sp^2^-to-sp^3^. These mechanistic postulates are affirmed in Kohn–Sham density functional calculations, which predict lower barriers for additive mechanisms for arene oxidation than H atom abstraction steps. The calculations show that the reaction proceeds on the pentadectet spin surface and that a non-innocent ligand participates in the transfer of the H atom. Following precedent literature which demonstrates that these Fe(IV)-oxo species react with C–H bonds in alkanes via hydrogen atom abstraction to form alcohols, it appears that iron(IV)-oxo species in MOFs exhibit duality in their reactions with inert hydrocarbon substrates akin to enzymes—if the C–H bonds are in saturated aliphatic hydrocarbons, then activation occurs via hydrogen abstraction, while if the C–H bonds are aromatic, then activation occurs by addition rearrangement.

## 1 Introduction

**Figure 1:**
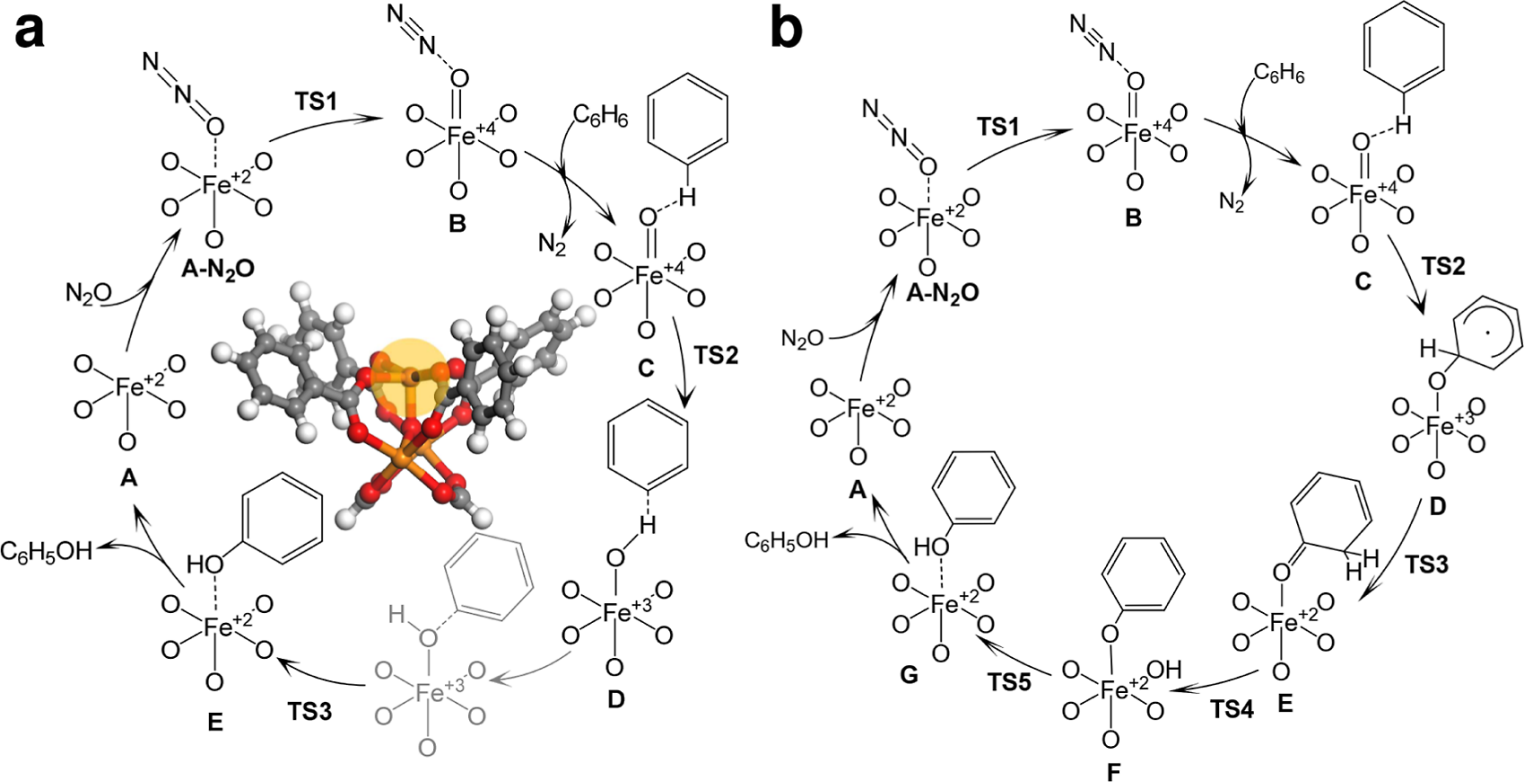
Schematics for the conversion of benzene to phenol on divalent Fe(II) sites in MIL-100(Fe) following (a) a hydrogen abstraction pathway followed by radical rebound (**habs**) or (b) an oxygen addition-rearrangement pathway without passing through the formation of benzene oxide (**addrear1**). Parts of the cycles considered in the present work are reported in black. In part (a), the structure of the **A** cluster is also reported, as optimized at the UM06-L/def2-TZVP level (2*S* + 1 = 15). Color code: red (oxygen), gray (carbon), orange (iron), and white (hydrogen). Reacting iron is highlighted with a yellow circle.

Metal–organic frameworks (MOFs) with reactive centers embedded in the framework provide an organized array of noninteracting, well-defined catalytic centers that are uniform in composition and placement.^1^ Following precedent literature in zeolites,^2,3^ Fe embedded in the MOFs, based on tri-iron oxo-centered metal nodes (e.g., MIL-100 and PCN-250, see inset in Figure 1a) has been shown to form open five-coordinated Fe(II) sites that react stoichiometrically with N_2_O to form Fe(IV)=O species similar to those reported for enzymes.^4−6^ These species can readily activate strong, apolar C–H bonds in short aliphatic hydrocarbons (CH_4_, C_2_H_6_, and C_3_H_8_) with the formation of the corresponding alcohols.^7−14^ Tri-iron oxo-centered cluster-based MOFs are also characterized by exceptional chemical and thermal stability.^15^ Additionally, simple and highly scalable synthetic protocols have been reported for these MOFs.^16^ Previously, we have reported^7,8^ that the so-formed high-spin open Fe(II) centers in Materials Institute Lavoisier (MIL)-100(Fe) and PCN-250(Fe) react with N_2_O to form high-valent Fe(IV)=O species, which are short-lived and highly reactive. Their high reactivity makes direct observation of Fe(IV)=O elusive, as shown by the in situ spectroscopic study performed by Xiao et al.^14^ on similar species in Fe_0.1_Mg_1.9_(dobdc)_2_. This Fe(IV)=O is an entatic state in which the weak ligand field induced by the carboxylate linkers and structural constraints induced by the MOF lattice result in a metal-oxo species that is facile at H atom abstraction from apolar C–H bonds in alkanes. The Fe(II) species formed upon thermal treatment of the MOF, which serve as the precursor to the Fe(IV)=O species, can be readily identified by Mössbauer, X-ray absorption, and NO probe molecule infrared adsorption spectroscopy, and the number of such sites can be enumerated by chemical titration under reaction conditions.^7,8^ The cleavage of aliphatic C–H bonds by Fe(IV)=O species proceeds in single-turnover events via hydrogen abstraction followed by radical rebound mechanisms^7−12,14^ as in heme and nonheme enzymes,^4−6,17^ resulting in the oxidation of CH_4_-to-CH_3_OH and of C_3_H_8_ to C_3_H_6_ and to C_3_H_7_OH. Partial oxidation products are formed with selectivity exceeding 60–75% at conversions exceeding 25% in stoichiometric reaction events, with the desired products of selective oxidation recovered upon washing the MOF postreaction.

Aromatic hydroxylation is another important class of reactions catalyzed by Fe(IV)=O sites in enzymes as a means of detoxification and excretion of toxic arenes.^18−21^ If a selective direct oxidation process for the conversion of benzene to phenol can be developed, it will eliminate the cosynthesis of acetone in the state-of-the-art industrial cumene process.^22^ Iron-based enzymes catalyze benzene hydroxylation using molecular oxygen as the oxidant in catalytic sequences initiated by electrophilic addition of high-valent iron-oxo species to π-bonds in benzene (step **C** to **D** in Figure 1b),^4,5,21,23,24^ without direct interaction with C–H bonds. Decay of the resulting σ-complex occurs in a manner that retains the original hydrogen of the activated C–H bond via migration of the substituent from the site of hydroxylation to the adjacent carbon, the so-called “NIH shift” (NIH from the US National Institute of Health that first reported on it, step **D** to **E** in Figure 1b).^25,26^ A similar mechanism has been proposed to occur on the extra-framework Fe centers in zeolites.^27^ However, to our knowledge, such a mechanism has not been demonstrated for abiotic heterogeneous catalysts containing Fe in the framework; in particular, no Fe-based MOFs have been reported to affect the direct oxidation of benzene to phenol using N_2_O as the oxidant.

Here, we demonstrate using a combination of Mössbauer spectroscopy, chemical titration, isotopic substitution, and Kohn–Sham density functional theory (DFT) calculations that Fe(II) species embedded in the framework of MIL-100 convert benzene to phenol with high selectivity (>95%) via electrophilic addition, akin to what is observed for enzymatic systems. MOFs thus provide an ideal platform for investigating electronic and structural factors in heterogeneous catalysts relevant to selective oxidation of recalcitrant C–H bonds.

## 2 Methods

### 2.1 Experimental Methods

#### 2.1.1 N_2_ Isotherms

The surface area of MIL-100(Fe) (STREM, USA) samples before and after the reaction was obtained by measuring N_2_ isotherms at 77 K using an ASAP 2020 surface area and porosity analyzer (Micrometrics). Samples were degassed at 423 K for 4 h prior to sample analysis, with surface areas determined by using the Langmuir model^28,29^ in the standard pressure range (0.05 < *p*/*p*_0_ < 0.20). All the reported quantities were affected by an error of 10%.

#### 2.1.2 Powder XRD

X-ray diffraction (XRD) patterns were collected in the 5–50° 2θ range in the Bragg–Brentano geometry using a laboratory diffractometer (Rigaku SmartLab SE) (step size of 0.01°, time per step 10 s). A Cu Kα anode (Kα, λ = 1.5406 Å) operated at 45 kV and 40 mA was used as the radiation source. The simulated patterns were obtained from previously reported cif files (Crystallography Open Database entry 7102029) and processed using Mercury software (Mercury 2022.1.0, CCDC).

#### 2.1.3 Mössbauer Spectroscopy

^57^Fe Mössbauer spectra were recorded at low temperatures (18 K) with samples cooled with liquid helium. Approximately 25 mg of the MIL-100(Fe) sample was mixed with 25 mg of boron nitride (Sigma-Aldrich). One sample was tested after reaction in benzene/N_2_O mixtures (2 and 90 kPa) at 523 K, with the sample sealed with Swagelok quick-connect fittings and transferred to a N_2_-filled glovebox to prevent air exposure. In the glovebox, the sample was mixed with boron nitride and loaded into a sample holder sealed with parafilm before being transferred to the spectrometer.

#### 2.1.4 Reaction Experiments

The reaction experiments were carried out in a recirculating batch reactor system with a reactor volume of 67 cm^3^. In a typical experiment, 53 mg of MIL-100(Fe) powder (STREM, USA) was loaded into a vertical 1/4 in. OD quartz reactor tube. The MOF was supported by plugs of quartz wool above and below the reactor bed and held in place by a 1/16 in. K-type thermocouple (Omega Engineering) from above and a 1/8 in. quartz rod at the bottom. The temperature in the reactor was controlled using the thermocouple, which provided a signal to the PID temperature controller (Watlow, EZ Zone), which in turn adjusted the power to an electric furnace (National Electric Furnace, FA 120, 120 V). A concentric copper block surrounding the reactor was used to minimize axial temperature gradients over the length. A schematic of the batch reactor set up with external recycle is shown in the Supporting Information (Section S8).

The MIL-100(Fe) sample was activated under vacuum (<3 Pa) at 523 K for 10 h, with the sample being heated from ambient temperature to 523 K at a rate of 0.033 K s^–1^, and subsequently the sample cooled to the reaction temperature (398 K). Mass flow controllers (instruments) were calibrated using a volumetric soap-film flow meter and used to control the flow of helium (Matheson, UHP Enable 99.999%), argon (Matheson, Research Purity 99.9999%), and nitrous oxide (Matheson, USP grade 99%). Benzene (Sigma-Aldrich, HPLC grade, 99.9%) was fed through a syringe pump (KD Scientific).

For the in situ NO titration experiments pursued to identify and count the number of accessible active sites, nitric oxide (Matheson, CP grade 99%) was injected into the reactor system through a dosing chamber (volume 0.1 cm^3^) through a 6-way valve (VICI Valco) prior to exposing MIL-100(Fe) to the reaction gas mixture of nitrous oxide and benzene.

The composition of the reactor effluent was quantified by gas chromatography (Agilent, 6890N) using a thermal conductivity detector and a flame ionization detector fitted with HP plot *Q* (Agilent) and CP Molsieve 5 A (Agilent) columns in series. The reactant gas mixture was recirculated at a rate of 1.17 cm^3^ s ^–1^ with a recirculation pump (Metal Bellows, MB-21). Online GC sampling was done to determine effluent gas compositions.

To terminate the reaction, the reactor was purged by flowing helium through the reactor bed at a rate of 0.83 cm^3^ s^–1^ for 30 min, and then the MOF was removed and washed with D_2_O (Cambridge Isotopes Limited) containing acetonitrile as an internal standard. The liquid containing MIL-100(Fe) was transferred to a centrifuge tube fitted with a 0.2 μm filter. The MOF sample was separated from the solution by using a microcentrifuge (operated at 6000 rpm). Desorbed products in solution were quantified using ^1^H NMR (Ascend 400, Bruker, 400 MHz, 64 scans, td 2 s).

#### 2.1.5 KIE Measurements

A mixture of ^13^C_6_H_6_ (Cambridge Isotope Limited) and ^12^C_6_D_6_ (Sigma-Aldrich) in a ratio of 1.47:1 was used to carry out the reaction. Benzene pressures in the range of 3–5 kPa were selected to maximize rates while still maintaining operation in the vapor phase (benzene vapor pressures ∼10 kPa at ambient temperature). The postreaction MOF sample was washed with H_2_O, and the products eluted in solution were analyzed with mass spectroscopy (Agilent technologies GC 7890B fitted with a 7200 QTOF-MS and DB-5 column) to determine the isotopologue distribution.

### 2.2 Computational Methods

#### 2.2.1 Density Functional Calculations

All calculations were performed using the Gaussian 16 program.^30^ The M06-L^31^ density functional in its unrestricted formalism (U) was used in combination with the def2-TZVP basis sets.^32,33^ This level of theory has been shown to accurately describe the electronic structure of single iron centers in ethane and methane oxidation studies^34^ and of the tri-iron oxo-centered cluster^9^ when compared to multireference wave function theory. Unfortunately, it overestimates by 30% the enthalpy of benzene oxidation with N_2_O to phenol and N_2_ with respect to the experimental value (−184 vs −261 kJ mol^–1^),^35^ in analogy with B3-LYP/def2-TZVP (−175 kJ mol^–1^), used in a similar study.^36^ The hybrid Minnesota functional M06 (27% Hartree–Fock exchange), although providing a better agreement, still overestimates the reaction enthalpy by 50 kJ mol^–1^. Nevertheless, the use of M06-L/def2-TZVP was dictated by its reliability in predicting the activation barrier of N_2_O activation for MIL-100(Fe) when benchmarked against experiments.^8^ For what concerns the present benzene-to-phenol reaction, this level of calculations predicts the kinetic isotope effect (KIE) in very good agreement with the experiments (see below), further validating the use of this level of calculations for modeling tri-iron oxo-centered-based MOFs. The cluster used to model the tri-iron metal node was carved from the periodic structure of the tri-iron oxo-centered MOF, MIL-127, optimized using the Perdew–Burke–Ernzerhof functional^37^ as implemented in the VASP 5.4.4 program,^38^ with long-range dispersion interactions considered using the scheme proposed by Grimme (D3).^39^ The cluster consists of the inorganic subunit of the MOF, terminated by four benzoate groups and two formate groups (Fe(III)_2_Fe(II)(μ_3_-O)(C_6_H_5_COO)_4_(COO)_2_, see Figure 1a). The four benzoate groups were placed around the reactive iron center to model eventual π–π interactions occurring between the substrate and the MOF. The positions of the C atoms of the phenyls were kept fixed to model the constraint associated with the presence of the MOF.

Geometry optimizations were carried out by means of the Berny optimization algorithm with an analytical gradient. A (99,590) pruned grid was used (i.e., 99 radial points and 590 angular points per radial point). Gaussian 16 default convergence thresholds were set for optimization. All the energetic data have been corrected for the basis set superposition error (BSSE) following the a posteriori method proposed by Boys and Bernardi,^40^ as implemented in Gaussian 16. The BSSE corrected values are indicated by a c superscript and were obtained from the computed *Y* values as *Y*^c^ = *Y* + BSSE.

Unscaled, harmonic vibrational frequencies were computed analytically. Enthalpies and Gibbs free energies were calculated at 1 atm and 298 K from a conventional ideal gas, rigid rotor, particle in a box, and quantum mechanical harmonic oscillator partition functions, except that the low vibrational frequencies (<50 cm^–1^) were replaced by a cutoff value (50 cm^–1^) following the De Moor scheme^41^ to account for limitations in the harmonic oscillator approximation for very low-frequency vibrations.^42−46^

Kinetic isotope effect studies for the replacement of all hydrogen atoms in benzene by deuterium atoms were evaluated using the approach discussed by De Visser and co-workers^47−49^ using the semiclassical Eyring model
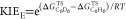
1where Δ*G*^TS^ is the free energy of activation, *R* is the gas constant, and *T* is the temperature (298 K). They also report a KIE value further corrected using the tunneling corrections reported by Wigner (KIE_W_)^47−49^ by multiplying KIE_E_ for the tunneling ratio
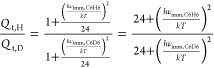
2

In this equation, *h* is Planck’s constant, *k* is Boltzmann’s constant, and ν_imm_ is the imaginary frequency in the **TS2** transition state.

## 3 Results and Discussion

Following our prior work for alkane activation on high-valent Fe-oxo species formed upon exposing tri-iron oxo-centered metal nodes in MIL-100(Fe) and PCN-250(Fe) to N_2_O,^7,8^ here we investigate the reactions of framework Fe(II) species in mixtures of N_2_O and benzene to determine reaction sequences for activation of recalcitrant arene C–H bonds with Fe(IV)=O species. The C–H bonds in benzene are even stronger than those in methane (472.5 kJ mol^–1^ versus 435 kJ mol^–1^ in CH_4_)^50^ and are not susceptible to direct electrophilic attack. However, unlike σ C–H bonds in methane, the C–H bonds in benzene are susceptible to oxidative addition because of the participation of aromatic π-orbitals in bonding to the metal-oxo species. Experimental and computational studies for benzene hydroxylation by the enzyme cytochrome P450^3,4,19−22^ note that high-valent iron oxyl species react with benzene via electrophilic addition to form a σ-complex based on an initial attack on the π-system of the benzene. This σ-complex decays to phenol via inter- or intramolecular mechanisms, retaining the hydrogen in the C–H that was activated, the so-called “NIH shift”. Here, we combine spectroscopic and kinetic experimental studies with DFT calculations to illustrate that Fe(IV)=O species in MOFs, similar to P450 enzymes,^5,21^ readily react with aromatic C–H bonds via oxidative addition pathways and selectively form phenol.

### 3.1 Material Characteristics and Reaction Scheme

**Figure 2:**
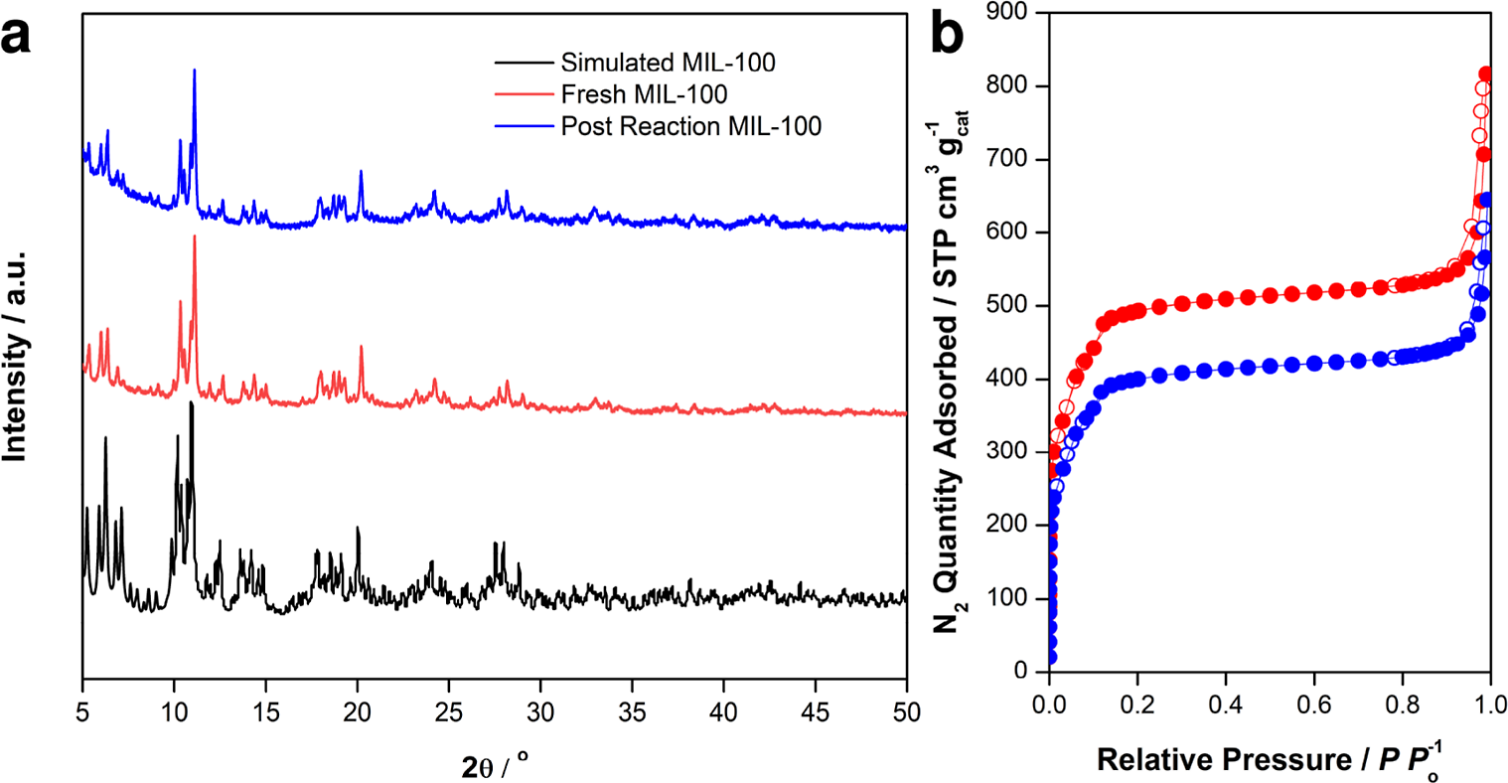
(a) PXRD patterns of MIL-100 as-received (red), after reaction (blue), and reference patterns (black) for MIL-100(Fe). Reaction conditions: the sample was activated at 523 K in vacuo for 10 h before exposure to 90 kPa N_2_O + 2 kPa C_6_H_6_ at 398 K for 2 h, followed by washing with D_2_O ex situ. (b) N_2_ isotherms of MIL-100(Fe) taken before (red curve, surface area (Langmuir) = 2106 m^2^ g^–1^) and after (blue curve, surface area (Langmuir) = 1729 m^2^ g^–1^) reaction. Samples were degassed at 513 K, and isotherms were collected at 77 K.

The MIL-100(Fe) sample was confirmed to be crystalline by its XRD pattern, which also the as-received material is the MIL-100 phase (see Figure 2a).^16,51^ The porosity of the material was determined by nitrogen adsorption at 77 K and is in line with previous reports (see Figure 2b).^7,8,16^ The structural integrity of the sample after the reaction was assessed by XRD and N_2_ adsorption studies, showing that the material retained high porosity (see Figure 2a,b). Further, the N_2_ isotherms do not evidence the formation of mesopores. The decrease in the surface area observed after the reaction (∼10%), although within the uncertainty of the measurement, can also be associated with impurities of the diluent used in the reactor (silica), which could not be completely eliminated from the postreaction sample. These characteristics mimic those we have reported in independent studies for MIL-100(Fe) samples that we used for examining reactions of N_2_O with CH_4_ and C_3_H_8_.^7,8^

**Figure 3:**
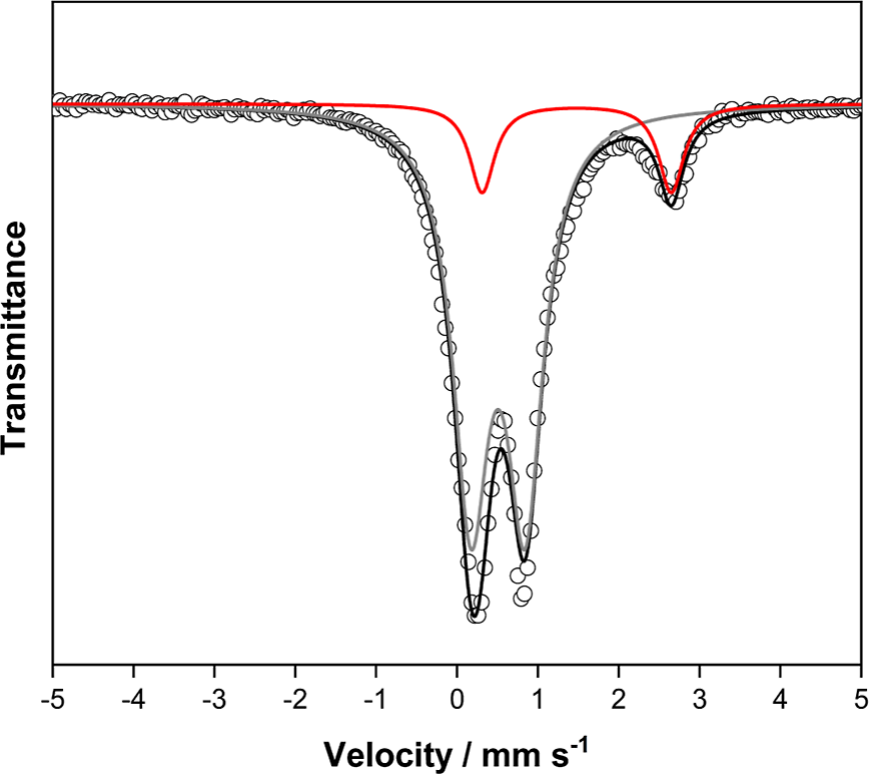
Mössbauer spectra of MIL-100 material treated to 513 K in vacuum and transferred to a spectrometer without exposure to the atmosphere. Spectra were collected at 18–20 K. Open circles—raw data, black line–fit to raw data curves for identified Fe species. Gray line—Lorentzian doublet assigned to an Fe(III) species (δ = 0.52 mm s^–1^, Δ*E*_Q_ = 0.67 mm s^–1^). Red line—Lorentzian doublet assigned to an Fe(II) species (δ = 1.5 mm s^–1^, Δ*E*_Q_ = 2.3 mm s^–1^).

The chemical characteristics of the Fe species in the MOF were probed by Mössbauer spectroscopy. MIL-100(Fe) samples undergo autoreduction upon thermal treatment when subjected to high temperatures (>473 K) in vacuo, notably resulting in the formation of high-spin Fe(II) sites.^7,8,15,52−55^ Accordingly, the Mössbauer spectrum of MIL-100(Fe) after thermal activation at 513 K in vacuum in Figure 3 clearly exhibits an additional doublet characteristic of high-spin (*S* = 2) Fe(II) species with an isomer shift of ∼1 mm s^–1^ and quadrupole splitting of ∼1.9 mm s^–1^. The presence of high-spin Fe(II) and Fe(III) species is in line with previous reports on MIL-100(Fe) and amorphous Fe-BTC.^15,52−54^

**Figure 4:**
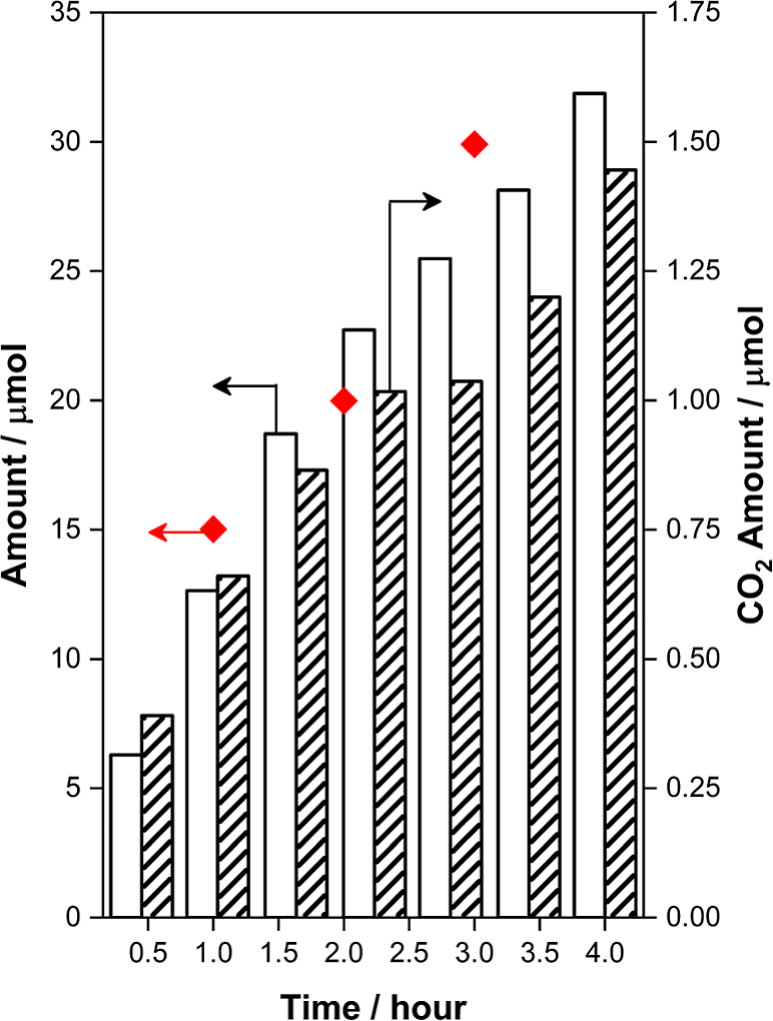
Yield of gas phase products in a batch reactor for a N_2_O + C_6_H_6_ experiment with 53 mg of MIL-100(Fe), 398 K, *P*_total_,_*t*=0_ = 110 kPa, *P*_N_2_O_,_*t*=0_ = 90 kPa, and *P*_C_6_H_6_,*t*=0_ = 2 kPa. All products were estimated by GC analysis, while phenol was estimated by NMR analysis after washing the MOF with D_2_O (striped bars: CO_2_, white filled bars: N_2_, and red diamonds: phenol).

Exposure of thermally activated MIL-100(Fe) to mixtures of N_2_O and benzene at 398 K in a recirculating gas phase batch reactor resulted in the production of N_2_ and trace amounts of CO_2_ in the gas phase (Figure 4). Individual reaction runs were quenched at durations of 1, 2, and 3 h. The MOF was washed with D_2_O ex situ, and the species present in the extract were quantified by ^1^H NMR. These data show that benzene-to-phenol oxidation occurs with ≥95% selectivity on a carbon basis and that N_2_O consumed to form N_2_ and Fe(IV)=O is quantitatively utilized to affect benzene-to-phenol conversion as opposed to oxidation of benzene to CO_2_. The present MOF shows a selectivity (>95%) mimicking that of enzymes and higher than that of iron-containing zeolites for prolonged times of reaction (<30%).^56^ An additional advantage is the absence of coke formation that affects the reaction catalyzed by iron-containing zeolites.^57^

**Figure 5:**
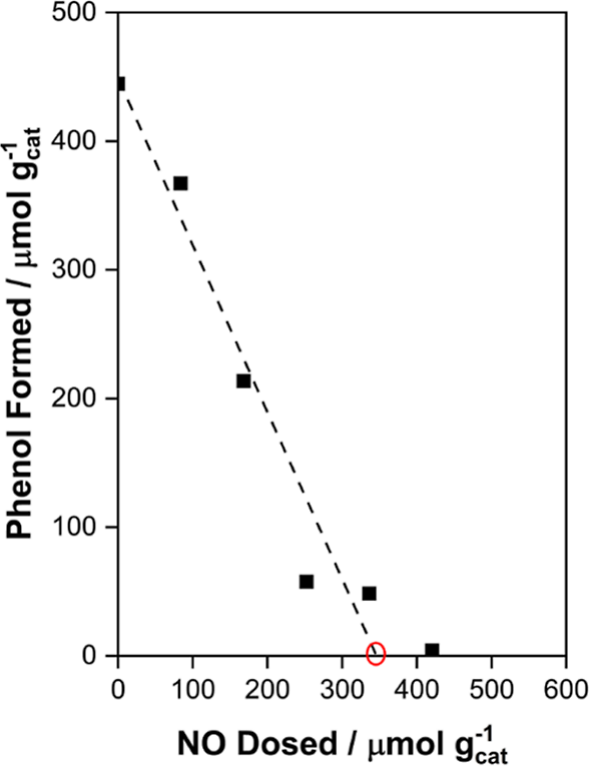
Moles of phenol formed (black squares) in the batch reactor as a function of NO dosed to the MOF before exposure to the reaction mixture (90 kPa of N_2_O and 2 kPa of benzene) at 398 K for 2 h. The estimated number of Fe(II) centers accessible under the reaction conditions is 340 μmol g_cat_^–1^ consistent with the estimate from Mössbauer analysis (about 325 μmol g_cat_^–1^, open red circle).

The relevance of Fe(II) species was probed in chemical titration studies similar to those we have reported previously for CH_4_ and C_3_H_8_ oxidation.^7,8^ Briefly, NO binds more strongly to Fe(II) than to Fe(III) species^2,55,58^ (108 vs 56 kJ mol^–1^ in MIL-100):^55^ if Fe(II) species are relevant for reaction, then the addition of NO should inhibit the rate. For chemical titration of benzene oxidation reactions, we performed a series of experiments in which a known amount of NO was preadsorbed on the MIL-100(Fe) after it was activated in vacuo at 473 K and, subsequently, measured the amount of phenol with ^1^H NMR after quenching the reaction and washing the sample with D_2_O. The results for this series of five independent titration experiments with varying amounts of preadsorbed NO are shown in Figure 5, noting that ∼340 μmol g_cat_^–1^ of Fe(II) is relevant for reaction, considering that each adsorbed NO can titrate one Fe(II) center. The amount of Fe(II) assessed from Mössbauer studies (∼325 μmol g_cat_^–1^) is also shown on this plot, to note the quantitative agreement between the Fe(II) concentration assessed from in situ chemical titration and ex situ Mössbauer studies.

Collectively, the structural and chemical characterization and the kinetic data discussed above allow us to infer that iron embedded in the framework of tri-iron oxo-centered MOFs is highly selective for single-turnover benzene hydroxylation reactions and these reactions involve Fe(II) species that react with N_2_O to form the oxidant. These experiments, however, do not allow us to infer whether H atom abstraction (**habs** in Figure 1a) or electrophilic addition to form a σ-complex (**addrear1** in Figure 1b and **addrear2** in Figure S1) occurs during the course of the reaction. Besides the signature “NIH shift” associated with arene oxidation by Fe(IV)=O species (**D** to **E** step in Figure 1b), another characteristic distinct for oxidative addition mechanisms for arene oxidation is the small or inverse kinetic isotope effect (KIE ≈ 1). KIE values of ∼0.9 were reported for benzene hydroxylation by nonheme iron-(IV)-oxo complexes [Fe(IV)(Bn-tpen)(O)]^2+^ and [Fe(IV)(N4Py)(O)]^2+^ by de Visser and co-workers,^47^ KIE values of 0.95–1.25 have been observed for the oxidation of deuterated chlorobenzenes,^59^ and KIE values of 1.04–1.16 were reported by Solomon and co-workers^27^ for benzene-to-phenol conversion on iron-containing zeolites by the Fe(IV)=O species associated with α-O in these materials. These KIE values preclude the occurrence of hydrogen-atom abstraction because KIEs for H atom abstraction mechanisms significantly exceed 1,^60,61^ and, notably, k_H_/k_D_ values of ∼1 suggest a small or inverse kinetic isotope effect in aromatic ring oxidation reactions, which would be consistent with a change in hybridization from sp^2^-to-sp^3^ occurring during the addition of an electrophilic iron-oxo group to the sp^2^ carbon in a benzene ring to form a σ-adduct.^47^ We performed a series of experiments to assess the KIE of benzene oxidation on MIL-100(Fe) materials to affirm whether electrophilic addition mechanistically describes benzene hydroxylation on iron-containing MOFs.

Mixtures of ^13^C_6_H_6_ (Cambridge Isotope Limited) and ^12^C_6_D_6_ (Sigma-Aldrich) in a ratio of 1.47:1 were reacted with N_2_O over MIL-100(Fe) in three independent experiments, varying the benzene partial pressure (3–5 kPa) while keeping the N_2_O pressure at 90 kPa. The products were extracted by washing the MOF with H_2_O. The KIE was determined from the isotopologue distribution of phenol as analyzed by mass spectrometry (Agilent technologies GC 7890B fitted with a 7200 QTOF-MS and DB-5 column) based on eqs 3–7. The KIE was assessed assuming first-order kinetics in benzene; the N_2_O reaction order does not need to be specified for this calculation
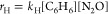
3
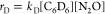
4
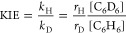
5where
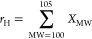
6
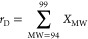
7

**Figure 6:**
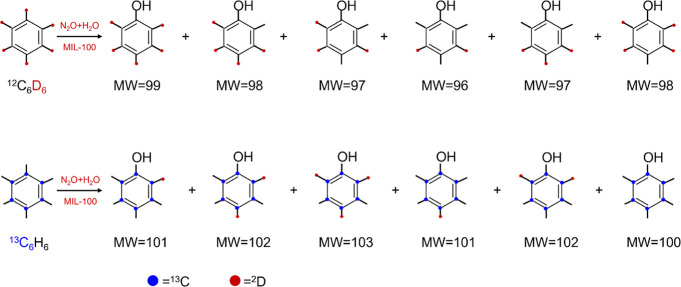
Schematic showing different phenol isotopologues resulting from ^13^C_6_H_6_ and ^12^C_6_D_6_ benzene.

MW is the molecular weight (MW is in g mol^–1^ and it is 94 = ^12^C_6_H_5_OH, 99 = ^12^C_6_D_5_OH, 100 = ^13^C_6_H_5_OH, and 105 = ^13^C_6_D_5_OH) and *X*_MW_ is the mole fraction for isotopologues with  MW estimated by MS analysis. The use of ^13^C_6_H_6_ and ^12^C_6_D_6_ precludes any potential overlap in mass of phenol isotopologues by H/D exchange upon washing with H_2_O (94 ≤ MW ≤ 99 and 100 ≤ MW ≤ 105, respectively, Figure 6, also see Section S7, Figures S6–S7, and Table S8).

The KIE values evaluated for each of the three independent experiments are reported in Table 1. An inverse KIE (KIE ≈ 0.85) is observed, which excludes the possibility of H atom abstraction and radical rebound mechanisms being relevant for arene hydroxylation. While the reaction order of benzene for benzene hydroxylation was not explicitly measured, an upper bound KIE of ∼1.25 can be determined if the hydroxylation reaction has zero-order kinetics in benzene. As a reference, we note that oxidation reactions that are known to transpire by hydrogen atom abstraction on nonheme Fe(IV)-oxo complexes are characterized by higher KIEs: 1.9 ≤ KIE ≤ 5.9 for the oxidation of methane to methanol in Fe-ZSM-5,^62^ while KIE values >30 have been reported for the oxidation of ethylbenzene to benzyl alcohol.^60,61^ The inverse KIE we assess is in line with an electrophilic oxidative addition mechanistic sequence that would result in a change from sp^2^-to-sp^3^ hybridization of the carbon center. In an effort to verify these mechanistic postulates, we pursued a complementary computational study using DFT to compare and contrast the possible modes of C–H activation in arene hydroxylation.

**Table 1:** KIE Estimated Based on Reactions of ^13^C_6_H_6_/N_2_O and ^12^C_6_D_6_/N_2_O after Exposure to 90 kPa N_2_O + 3–5 kPa (Benzene) at 398 K for 2 h, Followed by Washing with H_2_O Ex Situ^a^

benzene (C_6_H_6_) pressure	3 kPa	4 kPa	5 kPa
KIE	0.83	0.84	0.85

a The KIE values reported considering the reaction to be first-order with regard to the concentration of benzene.

### 3.2 Computational Studies

**Figure 7:**
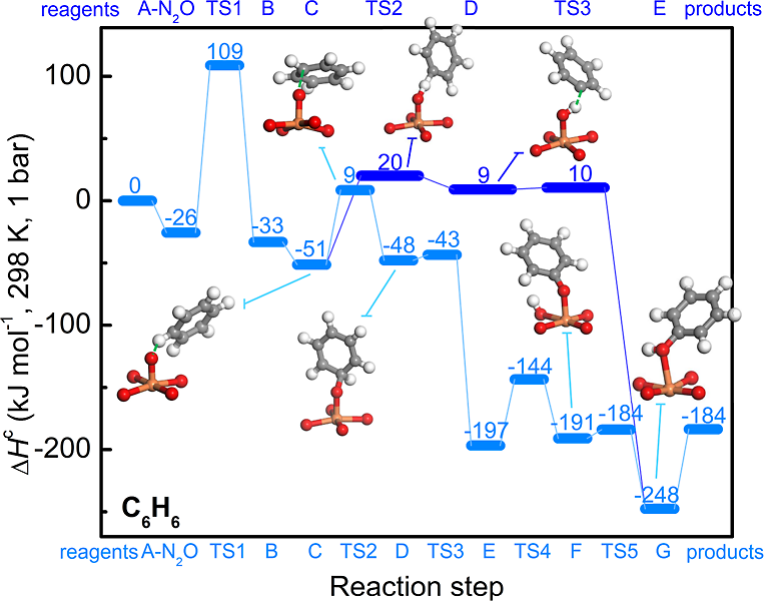
Energy profile reporting phenol formation on the metal node of MIL-100(Fe) through benzene oxidation by N_2_O. Reaction mechanisms: **habs** (blue) and **addrear1** (light blue). The **habs** mechanism requires the abstraction of hydrogen from benzene to form a benzyl radical. The **addrear1** pathway is an addition rearrangement mechanism supposing a non-innocent iron ligand that can shuttle a proton to the oxo group. A similar mechanism has also been proposed for other single iron catalysts, i.e., iron zeolites,^27^ enzymes,^21^ and nonheme iron complexes.^47^ Reaction profiles were calculated at the UM06-L/def2-TZVP level on the 2*S* + 1 = 15 spin surface. Enthalpies of the separated reactants (the **A** cluster, N_2_O, and one benzene molecule) have been set as zero. The optimized structure of the Fe center, its first coordination sphere, and the interacting species are shown for relevant steps. Color code: red (oxygen), gray (carbon), orange (iron), and white (hydrogen).

Two competitive pathways were considered in the DFT calculations: (a) hydrogen abstraction followed by radical rebound (**habs,** see Figure 1a) and (b) oxygen addition and rearrangement (**addrear1,** see Figure 1b). Both mechanisms share the formation of a ferryl species through the decomposition of N_2_O (**B**), followed by the coordination of benzene (**C**). The **habs** pathway has the following additional steps (see Figure 1a): (i) C–H bond activation with the formation of a benzyl radical (from **C** to **D**), (ii) formation of phenol through radical rebound (from **D** to **E**), and (iii) desorption of phenol, which corresponds to the regeneration of the MOF (from **E** to **A**). The mechanism for the **addrear1** pathway is similar to that reported for zeolites.^27^ After **C** formation, **addrear1** proceeds with (i) electrophilic attack of the oxoferryl on benzene with the formation of the σ-complex (**D**); (ii) hydrogen shift from the hydroxylation site to the adjacent carbon, with the formation of a ketone intermediate (“NIH shift”, from **D** to **E**); (iii) proton transfer from the dienone to the oxygens of the framework with the formation of phenolate (from **E** to **F**); (iv) reshuttling of the proton to the oxo group and formation of phenol (from **F** to **G**); and (v) desorption of phenol (from **G** to **A**). The corresponding reaction profiles of **habs** and **addrear1** are reported in Figure 7, while the corresponding numerical values are listed in Tables S3–S4.

Alternative to the formation of the σ-complex, oxygen addition can also pass through the formation of an arene oxide.^63,64^ Arene oxides are known to isomerize to phenols at physiological pH.^63^ Two alternative arene oxide pathways, including the formation of an epoxide as an intermediate, have been suggested in the literature,^20,21,63^ here indicated as **daad** and **addrear2** (Figure S4a). There, the benzene oxide is formed from **C** in the **addrear1** mechanism (see Figure 1b) through the insertion of the oxygen on the double bond (direct addition, see **dadd** in Figure S4a) or it is formed from intermediate **D** (**addrear2**, see Figure S4a). The **daad** mechanism has been excluded in both heme^21,63^ and nonheme^27,47^ single-iron-based catalysts. In tri-iron oxo-centered MOFs, the formation of benzene oxide (**C** → **E′** step) is exothermic by −28 kJ mol^–1^ (see Table S5), i.e., the reaction is favored thermodynamically. Nevertheless, we were not able to locate the transition state **TS2′** of the **daad** pathway, although a very large and diversified set of starting geometries was considered. The transition structure linking **C** with benzene oxide is a second-order saddle point, with two imaginary frequencies: one mode corresponds to oxygen addition and the second mode corresponds to the transition state corresponding to **E′** formation in **addrear2**. Based on these results, we can rule out the **daad** pathway also on MIL-100(Fe), similar to all single-atom iron-based catalysts reported so far.^65^ The formation of the epoxide in **daad** (**D’** → **E′** step) is slightly more exothermic than **C** → **E′** in the **addrear2** mechanism (−31 versus −28 kJ mol^–1^, respectively). The enthalpic barrier associated with transition state **TS3′** is 31 kJ mol^–1^. This means that benzene oxide can form during the reaction through the **addrear2** mechanism, along the lines of what has been previously reported from computational and experimental studies for heme^21,63^ and nonheme^47^ catalysts. Nevertheless, the competing **TS3** and **E** in the **D** → **E** reaction in **addrear1** are more stable than **TS3′** and **E’** (by 26 and 117 kJ mol^–1^, respectively). This means that benzene oxide can form eventually during the reaction through the **addrear2** mechanism, although the competing formation of dienone (**E** in **addrear1**) is decidedly more favored, both from a kinetic and thermodynamic vantage point.

In all the mechanisms considered in the computational study, the rate-determining step is associated with the formation of the ferryl species, as verified also for the cleavage of aliphatic C–H bonds on tri-iron oxo-centered metal nodes in MOFs.^7^ The second highest barrier is associated with the **C** → **D** step for both **habs** and **addrear1**, although it corresponds to different reactions in the two pathways: the C–H bond scission in **habs** and the oxygen addition for **addrear1**. The oxygen addition step is akin to that noted for other single Fe-based catalysts.^18,19,27,66^ The **C** → **D** step in **habs** is computed to be endothermic by 60 kJ mol^–1^ with an associated enthalpic barrier (Δ*H*_TS2_^c^) of 71 kJ mol^–1^. After the formation of the benzyl radical, the reaction proceeded in an almost barrierless fashion with the formation of phenol. For the competing **addrear1** pathway, the **C** → **D** step is computed to be only slightly endothermic (3 kJ mol^–1^) with a Δ*H*_TS2_^c^ of 60 kJ mol^–1^. Δ*H*_TS2_^c^ computed for MIL-100 is close to the one reported for a similar mechanism in P450 heme enzyme (73 kJ mol^–1^),^21^ while it is higher than in nonheme Fe(IV)-oxo species in [Fe(IV)(N4Py)(O)]^2+^ (36 kJ mol^–1^)^47^ and in Fe-BEA* (barrierless).^27^

In the so-formed σ-complex on MIL-100 (**D** in **addrear1**), the substrate is a radical (see Table S1), and it shares very similar electronic and geometric features with σ-complexes having a radical character formed on other single iron-based catalysts.^8,10,18^ The comparison of the α-LUMO orbitals for **C** and **D** (see Figure S2) provides evidence that this step, as expected, corresponds to the donation of an electron from benzene to the molecular orbital, having a main contribution from the 3d_z_^2^ orbital of iron. The reaction then proceeds with the formation of 2,4-cyclohexadienone through an NIH shift (**E**). This dienone intermediate is significantly stabilized with respect to **D** by −149 kJ mol^–1^. Correspondingly, the NIH-shift barrier is very low (5 kJ mol^–1^, **TS3**). The change of the β-HOMO from **D** to **TS3** and from **TS3** to **E** is reported in Figure S3. The reaction then proceeds via the formation of a phenolate (**E** → **F**) species through a proton-shuttle mechanism mediated by the framework oxygens. This step is slightly endothermic and has an associated barrier of 53 kJ mol^–1^. The proton is then reshuffled back to the substrate (**F** → **G**) with the formation of phenol. In this case, the process is associated with a barrier of only ∼7 kJ mol^–1^. The direct involvement of the first coordination shell of iron in the reaction makes it a non-innocent ligand. The cluster oxygens mediate the proton shuttle, enabling the transformation of the surface-bound benzene oxide to phenol. It is noteworthy that Fe species embedded in abiotic heterogeneous frameworks share this detail of the mechanism with completely different classes of catalysts for benzene hydroxylation, having been reported for enzymes,^21^ nonheme Fe(IV)-oxo species,^47^ and zeolites (Fe-BEA*).^27^ This common behavior is surprising because these compounds, besides the presence of Fe(II) centers, do not share a common chemical composition, dimensionality, or structure and do not have similar accessibility to the metal centers to incoming adsorbates. This finding suggests that Fe(II) centers exhibit common behavior in C–H bond oxidation, independent of the structure that hosts them.

Thus, the results from our calculations noting lower activation and reaction enthalpies for the **addrear1** pathway than the **habs** pathways agree with the experiments pointing toward the electrophilic addition mechanism as kinetically and thermodynamically favored over one involving hydrogen abstraction. Further supporting this assignment, the computed KIE values for the **addrear1** pathway are very close to the experimental values (0.93 and 0.72 for KIE_E_ and KIE_W_, respectively, versus ∼0.85 for the experimental KIE assuming first-order kinetics in benzene). In contrast, the computed KIE_E_ for the **habs** pathway is 5.12, which is greater than the upper bound KIE of ∼1.25 assuming zero-order kinetics in benzene. The similar KIE values assessed from experiments and computational studies suggest that single iron centers present in the MOFs affect benzene hydroxylation through electrophilic substitution, in analogy with enzymes,^21^ homogeneous catalysts,^47^ and extraframework iron species in zeolites.^27,62^ It is evident from this report and from precedent literature on enzymes and zeolites that Fe(IV)-oxo species exhibit duality in mechanism depending on the nature of the C–H bond to be oxidized, independent of the chemical composition, structure, morphology, or size of the catalyst. Specifically, Fe(IV)-oxo species react with aliphatic C–H bonds via hydrogen atom abstraction to form alcohols, while they activate aromatic C–H bonds by addition-rearrangement. The results of this study allow us to highlight the ability of ferryl species to maintain this characteristic also for a class of Fe-MOFs. This particular characteristic can only be partially attributed to the higher dissociation energy of the Fe(IV)-oxo bond with respect to the other iron ligands because the **addrear** mechanisms require the direct involvement of the ligand. The generality of this observation for MOFs as well as the specific energetic drivers for this duality of C–H bond activation in alkanes and aromatics should be addressed in future studies.

The last step of the reaction is the desorption of phenol to restore the open Fe(II) site (**G** → **A**). This process is endothermic by 64 kJ mol^–1^ (see Figure 7). Phenol binding enthalpy is then large enough to “poison” the Fe(II) centers, being larger than the binding enthalpy of benzene (−33 kJ mol^–1^) and N_2_O (−26 kJ mol^–1^) to the Fe(II) sites. In analogy with other catalysts,^36,57,67^ this could explain the >95% selectivity observed in the experiments, wherein the bound form of phenol avoids its overoxidation.

**Figure 8:**
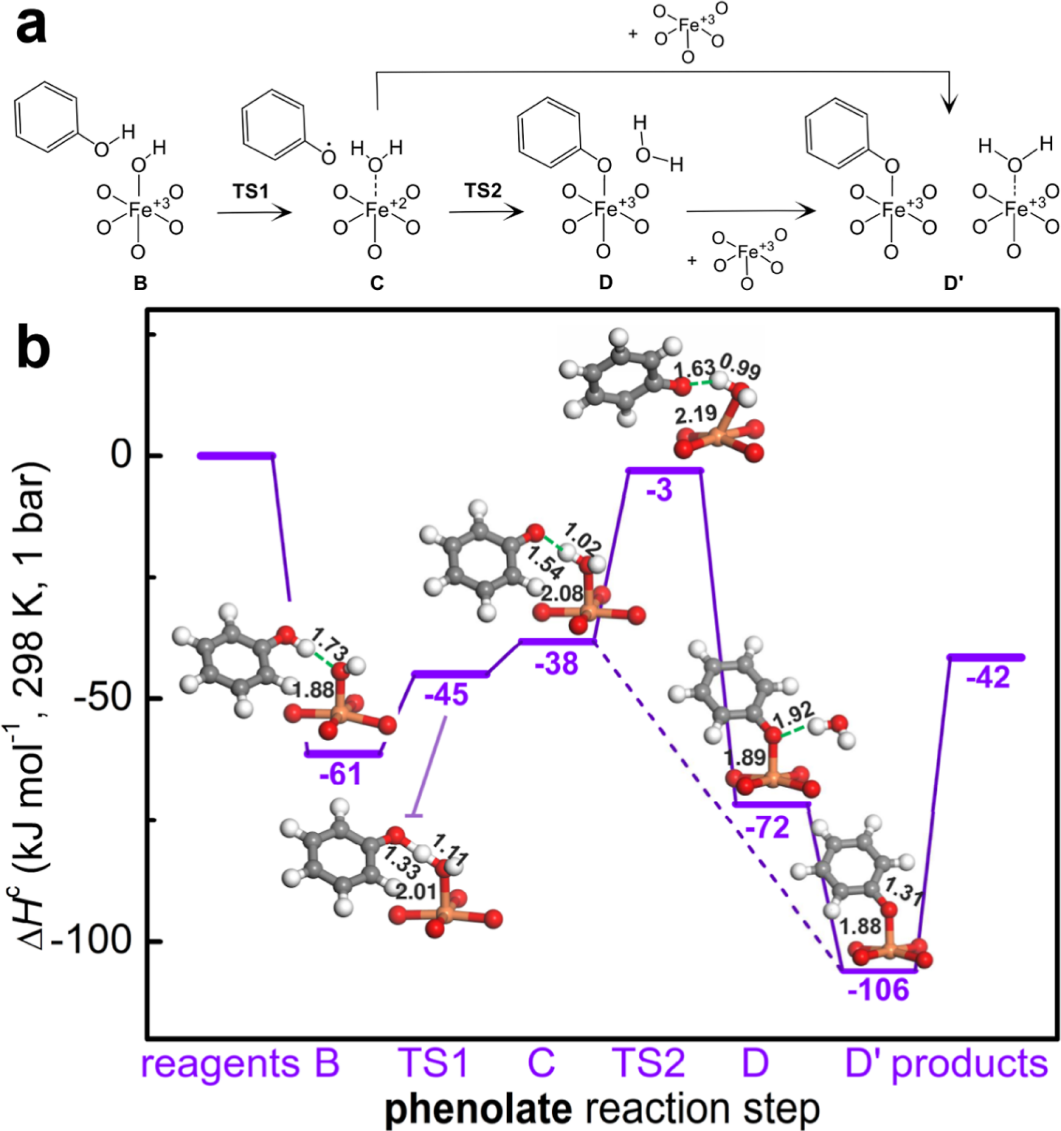
Phenoxy formation from phenol over Fe(III)–OH sites in MIL-100(Fe). (a) Schematics for phenol dehydration via the Fe–O scission pathway. (b) Reaction enthalpy profiles for Fe–O scission dehydration pathways calculated at the UM06-L/def2-TZVP level on the 2*S* + 1 = 16 spin surface. Enthalpy of the separated reactants (the hydroxylated form of the **A** cluster and one phenol molecule) was set as zero enthalpy. The optimized structure of the Fe center, its first coordination sphere, and interacting species is shown for each step. Color code: red (oxygen), gray (carbon), orange (iron), and white (hydrogen).

Protection mechanisms are known to be key in maintaining high selectivity in the methane to methanol reaction and avoiding methanol overoxidation.^6,7,68−70^ The formation of stable methoxy species hinders the diffusion of methanol in the gas phase, thus avoiding further oxidation. These methoxy species can be recovered in the form of methanol only by washing the sample postreaction in water. A protection mechanism through the formation of phenolates could explain both the high selectivity and the high thermal stability of the phenol in the MOF. Methoxy species can readily form on acidic surfaces such as zeolites^42−45^ and polyoxometalates,^46^ through single-step^44,45^ or two-step dehydration mechanisms.^42^ The activation barriers reported for supports with strong Brønsted acid sites range from 230 kJ mol^–1^ in SAPOs^44^ to 80 kJ mol^–1^ in ZSM-22.^42^ Simons et al.^7^ have recently shown that Fe–OH species formed during the reaction on tri-iron oxo-centered clusters in MOFs allow a similar methanol protection mechanism, although here methoxy species are formed through the deprotonation of methanol because of the low Brønsted acidity of the Fe–OH species. The activation enthalpy of this process in PCN-250 and MIL-100(Fe) is computed to be lower than what is reported for acid surfaces (57 kJ mol^–1^).^7^ We have verified that these Fe–OH species can react with phenol following a mechanism similar to that of methanol, resulting in the formation of Fe-phenolates. The feasibility of this phenol protection mechanism is evident from the DFT reaction profiles reported in Figure 8. The activation barrier for the formation of phenolate by deprotonation of phenol (**B** to **C**) is computed to be only 23 kJ mol^–1^, i.e., about a third of the activation enthalpy reported for methoxy formation (57 kJ mol^–1^). The reaction then proceeds downhill with the coordination of phenolate by an open Fe(III) center (**C** to **D′**) or through the displacement of water by the phenolate (**C** to **D**) with an activation barrier of 35 kJ mol^–1^. Mechanisms for the formation of phenolate from the reaction of phenol with oxygenated iron complexes in Fe-ZSM-5 have also been proposed by Li et al.,^57^ with activation energies ranging from 19 to 65 kJ mol^–1^. This result suggests that deprotonation of hydroxylated species is likely a general protection mechanism in the presence of surface –OH species, independent of the aliphatic or aromatic nature of the substrate. It is important to stress that the formation of Fe-phenolates causes the poisoning of the iron centers while concurrently enhancing the selectivity of the reaction. Phenolates have been suggested to be coke precursors in Fe-ZSM-5.^57^ Although a large amount of phenolates are formed in MIL-100(Fe), coke formation was not observed. The absence of coking in MOFs can be associated either with the mononuclearity of Fe sites (not guaranteed in zeolites) or with the lower basicity of the framework oxygen atom in MOFs, which, in turn, limits phenolate formation to the mechanism reported in Figure 8 and excludes coupling/dehydrogenation of more than one phenol molecule per iron site in the MOF.

## 4 Conclusions

Fe(II) species embedded in tri-iron oxo-centered clusters of MOFs affect the selective hydroxylation of benzene to phenol in stoichiometric reactions with N_2_O. Structural and chemical characteristics of the MIL-100(Fe), probed before and after the reaction using a combination of N_2_ uptake, XRD, and Mössbauer spectroscopy, suggest that the material retains its porosity and crystallinity over the course of the reaction and that Fe(II) sites are engendered upon thermal treatment of the MOF. The involvement of Fe(II) sites in benzene hydroxylation is affirmed in chemical titration studies with NO, with rates of benzene hydroxylation being entirely suppressed at NO uptakes of ∼340 μmol g_cat_^–1^. Benzene hydroxylation appears to proceed via electrophilic addition, mimicking enzymes, as evidenced by the inverse or small kinetic isotope effect assessed experimentally and verified in Kohn–Sham DFT calculations. Electrophilic addition mechanisms that result in arene hydroxylation on framework Fe-based MOFs are distinct from radical rebound mechanisms that result in aliphatic hydroxylation of aliphatic C–H bonds on these same MOFs. However, the mechanisms that protect the desired product from being overoxidized and that involve dehydrogenation on Fe(III)–OH groups to form persistent surface-bound oxo-species are common for alkane and arene oxidation on Fe-based MOFs. These results suggest that Fe species embedded in microporous materials provide a platform for the design of novel catalysts for the selective hydroxylation of arenes.

## Supporting Information Available

Mass spectrometric analysis data for evaluation of kinetic isotope effects; relevant geometrical, electronic, and energetic parameters for all the structures; and additional energetic plots (PDF)
Cartesian coordinates for the all the optimized geometries (ZIP)

## Abbreviations

MIL: Materials Institute Lavoisier
IR: infrared spectroscopy
KS-DFT: Kohn–Sham density functional theory
MOFs: metal–organic frameworks
