# Supplementary material for: Mechanism of Benzene Hydroxylation on Tri-Iron Oxo-Centered Cluster-Based Metal–Organic Frameworks
Source: J Phys Chem C Nanomater Interfaces. 2023 Nov 24;127(48):23246–57. doi: 10.1021/acs.jpcc.3c06423 (PMC10711795; doi:10.1021/acs.jpcc.3c06423)
Supplement: Supplementary file 1 — jp3c06423_si_001.pdf [file jp3c06423_si_001.pdf]

Supporting Information

Mechanism of Benzene Hydroxylation on Tri-Iron Oxo-Centered Cluster-Based Metal-Organic Frameworks

Jenny G. Vitillo,[a] Madhuresh Choudhary,[b] Matthew C. Simons,[b] Laura Gagliardi,[c] Aditya Bhan[b]**

[a]Department of Science and High Technology and INSTM, Università degli Studi dell'Insubria, Via Valleggio 9, I-22100 Como, Italy.

[b]Department of Chemical Engineering and Materials Science, University of Minnesota, 421 Washington Avenue S.E., Minneapolis, Minnesota 55455, United States.

[c]Department of Chemistry, Pritzker School of Molecular Engineering, James Franck Institute, University of Chicago, Chicago, IL 60637, United States.

*e-mail: jg.vitillo@gmail.com

*e-mail: abhan@umn.edu

Table of Contents

S1. Additional Computational Details 2

S2. Additional Data on the Clusters in addrear1 Mechanism 3

S3. Benzene Oxide Mechanisms 5

S4. Additional Data on the Clusters in addrear2 Mechanism 6

S5. Energetic parameters of the clusters 7

S6. Additional Energetic Parameters 10

S7. Mass Spectrometric Data for Evaluation of Kinetic Isotope Effects 11

S8. Reactor Unit set-up 13

References 14

S1. Additional Computational Details

Cartesian coordinates of all the optimized structures are reported in the Supplementary Material and at the Zenodo repository with DOI: 10.5281/zenodo.6569449. All the relevant energetic parameters are reported in Tables S1-S5.

Spin-ladder computations for the metal cluster predict that the iron in $[Fe(III)_2Fe(II)(\mu_3\text{-}O)]^{6-}$ units is in a high-spin state, in agreement with the Mössbauer spectra.[1] The antiferromagnetic coupling between two Fe(III) atoms within the nodes, considered in the "broken symmetry" solution (BS), is computed to be more stable than the fully coupled high-spin state (HS). For systems having a high multireference character as the metal node of MIL-100(Fe), more accurate energies are obtained using the BS solution, although the wave function is not a spin eigenfunction nor does it have the correct spin density.[2] Moreover, following a reaction profile on a broken symmetry surface can be cumbersome,[2, 3] also hindering the reproducibility the results. The difference in energy between the HS and BS solution is of only 20 kJ mol^{-1}. For this reason, we report all the mechanisms obtained for HS ($2S + 1 = 15$ for the phenol formation and $2S + 1 = 16$ for phenolate formation). Please refer to Refs. 2-4 for a detailed discussion on this choice in general[2-4] and for the triiron-oxo centered MOFs, in particular.[3]

The **A-H** cluster used in the mechanism for phenolate formation in shown in Figure S1.

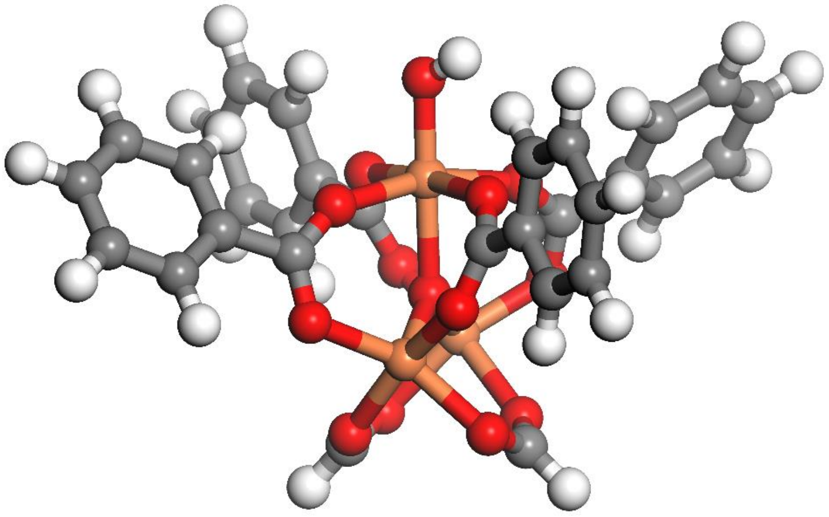

Figure S1. Hydroxylated form of the cluster model used for the tri-iron oxo-centered metal node. Structure of the **A-H** cluster as optimized at the UM06-L/def2-TZVP level ($2S + 1 = 16$). Color code: red (oxygen), grey (carbon), orange (iron), white (hydrogen).

S2. Additional Data on the Clusters in addrear1 Mechanism

In Table S1, electronic and geometrical parameters for the catalyst along the **addrear1** pathway are reported.

Table S1. Spin densities ρ and charges q on the reacting Fe, on the oxygen involved in the hydroxylation reaction O and on the 6 C and 6 H atoms originating from the benzene molecule for all the clusters in the **addrear1** pathway as obtained at the UM06-L/def2-TZVP level on the ($2S + 1 = 15$) spin state. The distance of O from the reacting iron center and the reacting C are also reported, d(Fe-O) and d(C-O) (in Å), respectively. Spin densities are expressed as the difference between the α and β electron densities.

**addrear1**	q(Fe)	q(O)	ρ(Fe)	ρ(O)	ρ(C)	ρ(6C6H)	d(Fe-O)	d(C-O)
A	0.86	–	4.01	–	–	–	–	–
A-N_2O	0.84	-0.04	4.03	0.02	–	–	2.437	–
TS1	0.86	-0.28	4.05	-0.10	–	–	1.870	–
B	0.89	-0.41	3.18	0.55	–	–	1.621	–
C	0.90	-0.40	3.18	0.54	0.00	0.01	1.623	3.333
TS2	0.86	-0.41	3.87	0.18	0.01	-0.42	1.736	1.839
D	0.88	-0.40	4.10	0.26	-0.01	-0.68	1.850	1.456
TS3	0.85	-0.36	4.07	0.09	0.01	0.03	2.042	1.366
E	0.89	-0.41	4.12	0.26	-0.01	-0.62	1.862	1.366
TS4	0.88	-0.34	4.09	0.11	0.01	0.04	2.005	1.273
F	0.88	-0.38	4.08	0.20	0.01	0.09	1.895	1.308
TS5	0.88	-0.37	4.07	0.10	0.00	0.05	2.057	1.338
G	0.85	-0.35	4.05	0.04	0.00	0.01	2.247	1.377
D-P450[a]	–	–	1.84	–	–	-0.85	1.802	1.500
D-P450[b]	–	–	1.06	–	–	0.46	1.882	1.482
D-N4PyFe[c]	0.87	-0.52	4.09	0.40	–	-0.87	1.792	1.530
D-N4PyFe[d]	0.57	-0.5	3.8	0.06	–	0.03	1.963	1.363
D-FeBEA[e]	0.99	-0.38	3.98	0.36	-0.01	-0.78	1.747	1.456

[a] P450: data from Ref. 5 for the radical form of the σ-complex (^{2}**2** intermediate, $2S + 1 = 2$).[b] P450: data from Ref. 5 for the cationic form of the σ-complex (^{2}**3** intermediate, $2S + 1 = 2$). [c] $[Fe(N4Py)(O)]^{2+}$: data from Ref. 6 for the radical form of the σ-complex (^{5}**B** intermediate, $2S + 1 = 5$). [d] $[Fe(N4Py)(O)]^{2+}$: data from Ref. 6 for the cationic form of the σ-complex (^{5}**A** intermediate, $2S + 1 = 5$). [e] Fe-BEA* system: single point on the structure for the σ-complex in Ref. 7 (**2** intermediate, $2S + 1 = 5$).

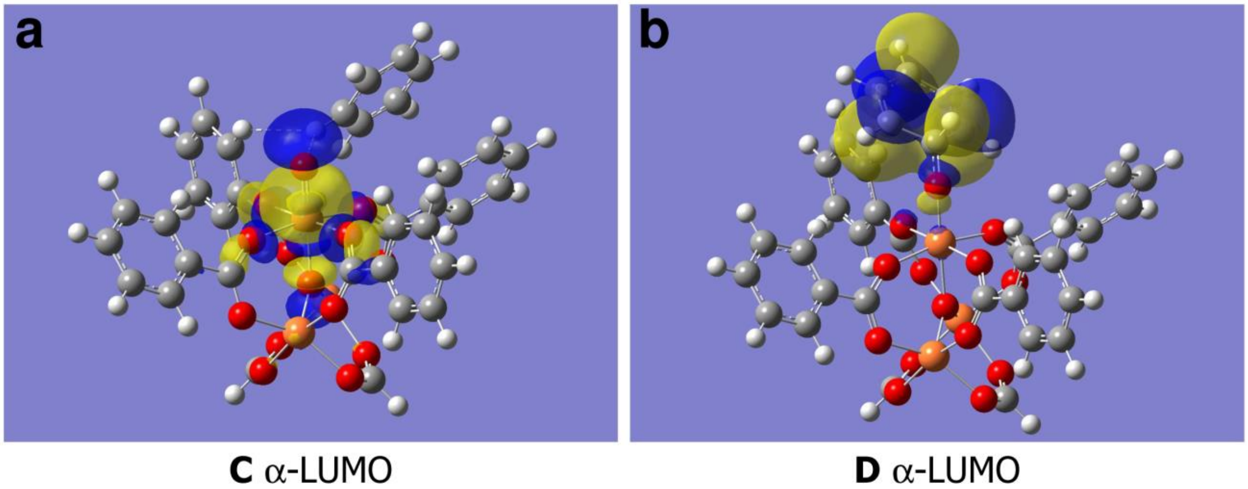

Figure S2. The lowest unoccupied molecular orbital along the C-O bond formation in the addrear1 pathway. α-LUMO orbital for **C** and **D** structures, as computed at the UM06-L/def2-TZVP level (2*S* + 1 = 15). Blue surfaces correspond to positive values, while yellow surfaces to negative values. Same color code for atoms as in Figure S1.

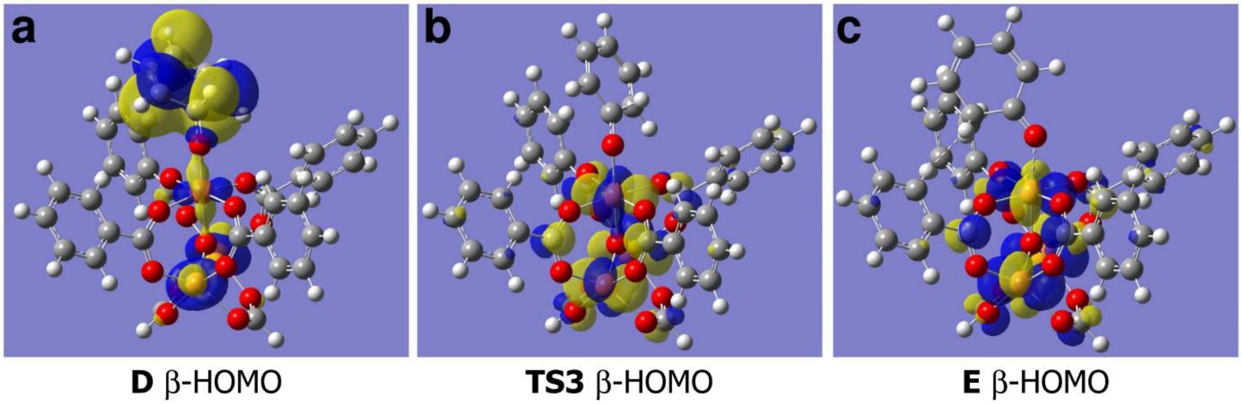

Figure S3. The highest occupied molecular orbital along the NIH shift step in the addrear1 pathway. β-HOMO orbital for **D**, **TS3**, and **E** structures, as computed at the UM06-L/def2-TZVP level (2*S* + 1 = 15). Blue surfaces correspond to positive values, while yellow surfaces to negative values. Same color code for atoms as in Figure S1.

S3. Benzene Oxide Mechanisms

Two additional mechanisms have been considered for the formation of phenol, that consider benzene oxide as an intermediate: **daad**, where the benzene oxide is formed from **C** of the **addrear1** mechanism through the insertion of the oxygen on the double bond (see **dadd** arrow in Figure S4), **addrear2**, where benzene oxide is formed from intermediate **D'** (see Figure S2).

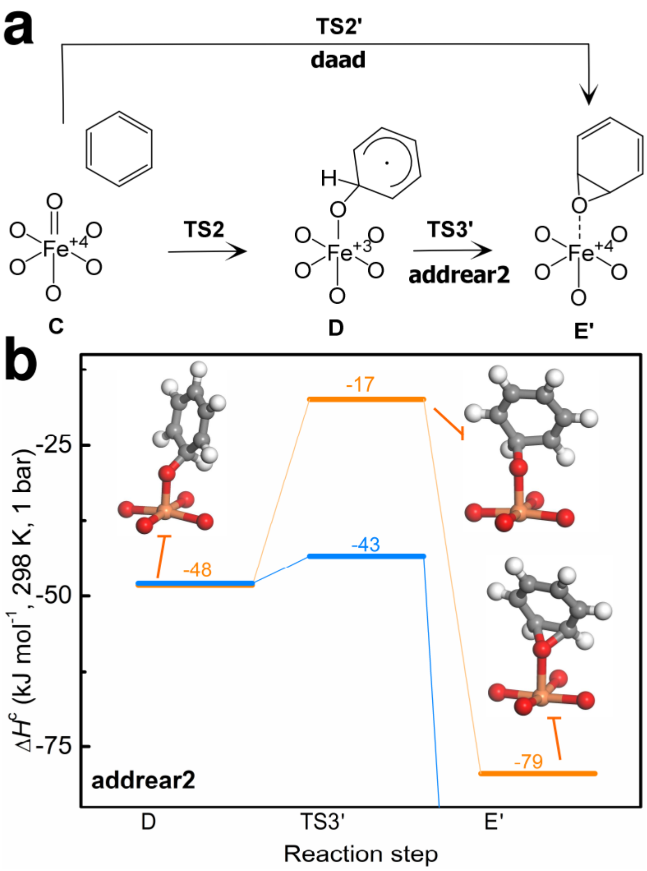

Figure S4. Mechanisms considering benzene oxide as an intermediate. Benzene oxide formation through (a) oxygen direct addition (**daad**) or by rearrangement of the σ-complex (**D** in **addrear1**, see Figure 1b) to benzene oxide (**addrear2** mechanism). (b) Reaction profile for **addrear2** (orange line) as computed at the UM06-L/def2-TZVP level ($2S + 1 = 15$). The corresponding step in **addrear1** is also shown for the sake of comparison (light blue). The optimized structure of the Fe center and its first coordination sphere, and the interacting species is shown for relevant steps of the **addrear2** pathway. Same color code for atoms as in Figure S1.

S4. Additional Data on the Clusters in addrear2 Mechanism

In Table S2, electronic and geometrical parameters for the catalyst along the **addrear2** pathway are reported. A σ-complex having a slightly different geometry than **D** was used in **addrear2** (**D’**) because it was possible to locate **TS3’** starting from **D’** while it was not possible starting from **D** (see Figure S5). **D’** is also energetically and electronically very similar to **D**, being more stable than **D** of < 1 kJ mol^{-1}.

Table S2. Spin densities ρ and charges q on the reacting Fe, on the oxygen involved in the hydroxylation reaction O and on the 6 C and 6 H atoms originating from the benzene molecule for all the clusters in the **addrear2** pathway as obtained at the UM06-L/def2-TZVP level on the ($2S + 1 = 15$) spin state. The distance of O from the reacting iron center and the reacting C are also reported, *d*(Fe-O) and *d*(C-O) (in Å), respectively. Spin densities are expressed as the difference between the α and β electron densities.

**addrear2**	*q*(Fe)	*q*(O)	*ρ*(Fe)	*ρ*(O)	*ρ*(C)	*ρ*(6C6H)	*d*(Fe-O)	*d*(C-O)
**D’**	0.88	-0.40	4.11	0.26	-0.01	-0.63	1.866	1.386
**TS3’**	0.87	-0.32	4.07	0.12	0.00	-0.27	1.955	1.429
**E’**	0.86	-0.20	4.06	0.05	0.00	0.02	2.183	1.442

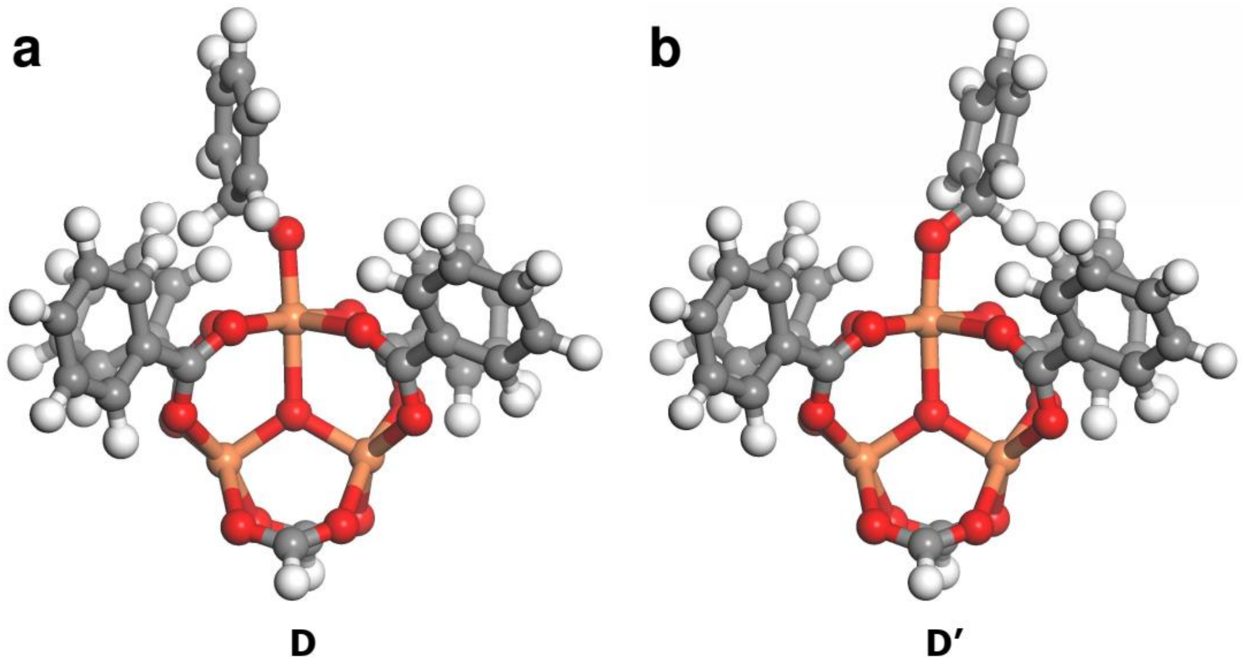

Figure S5. Comparison between D-addrear1 and D’-addrear2. Structures as optimized at the UM06-L/def2-TZVP level ($2S + 1 = 15$). Color code: red (oxygen), grey (carbon), orange (iron), white (hydrogen).

S5. Energetic parameters of the clusters

In the following tables, the computed electronic, enthalpy and free Gibbs energy for all the intermediates and transition states are provided for the **habs**, **addrear1**, and **addrear2** (reported in Figure 1a, Figure 1b, and Figure S4). The values computed for the phenolate formation mechanism (reported in Figure 3) are shown in Table S6.

Table S3. Electronic energy, enthalpy, and Gibbs free energy for all the intermediates and TS structures in the **habs** mechanisms reported in Figure 1a related to the formation of phenol through the hydrogen abstraction from a benzene molecule on a tri-iron oxo-centered metal node, as obtained at the UM06-L/def2-TZVP level on the pentadectet spin surface. These energies are reported as absolute values (E, H^0, G^0, in hartree) or referenced to the energies of the separated reagents (**A**, N_2O, and benzene) and corrected for BSSE (ΔE^c, ΔH^{0c}, ΔG^{0c}, in kJ mol^{-1}; for the electronic energy, the value not corrected for BSSE is also reported for comparison, ΔE). H^0 and G^0 have been calculated at 1 atm and 25 °C. The imaginary frequency $\tilde{\nu}_{imm}$ for each TS is also shown (in cm^{-1}).

**habs**	E	H^0	G^0	ΔE	ΔE^c	ΔH^{0c}	ΔG^{0c}	$\tilde{\nu}_{imm}$
**A**	-5926.50816949	-5926.135719	-5926.224777	–	–	–	–	–
**A-N2O**	-6111.25478659	-6110.865731	-6110.964076	-32.7	-29.6	-25.5	15.4	–
**B**	-6111.19963007	-6110.814515	-6110.913437	112.1	115.1	108.8	148.2	–
**TS1**	-6111.25561000	-6110.867844	-6110.968931	-34.8	-33.8	-33.1	0.6	-643
**C**	-6234.00028178	-6233.514966	-6233.620189	-57.1	-52.8	-51.2	0.6	–
**TS2**	-6233.96410011	-6233.487541	-6233.592742	37.9	41.5	20.1	72.0	-1659
**D**	-6233.97477919	-6233.491731	-6233.600016	9.9	13.4	9.1	52.9	–
**TS3**	-6233.97250563	-6233.491093	-6233.597453	15.8	19.2	10.6	59.4	-175
**E**	-6234.07671091	-6233.590582	-6233.694711	-257.7	-251.3	-247.6	-192.9	–

Table S4. Electronic energy, enthalpy, and Gibbs free energy for all the intermediates and TS structures in the **addrear1** mechanisms reported in Figure 1a related to the formation of phenol through electrophilic addition from a benzene molecule on a tri-iron oxo-centered metal node, as obtained at the UM06-L/def2-TZVP level on the pentadectet spin surface. These energies are reported as absolute values (E, H^0, G^0, in hartree) or referenced to the energies of the separated reagents (**A**, N_2O, and benzene) and corrected for BSSE (ΔE^c, ΔH^{0c}, ΔG^{0c}, in kJ mol^{-1}; for the electronic energy, the value not corrected for BSSE is also reported for comparison, ΔE). H^0 and G^0 have been calculated at 1 atm and 25 °C. The imaginary frequency $\tilde{\nu}_{imm}$ for each TS is also shown (in cm^{-1}).

**addrear1**	E	H^0	G^0	ΔE	ΔE^c	ΔH^{0c}	ΔG^{0c}	$\tilde{\nu}_{imm}$
**A**	-5926.50816949	-5926.135719	-5926.224777	–	–	–	–	–
**A-N2O**	-6111.25478659	-6110.865731	-6110.964076	-32.7	-29.6	-25.5	15.4	–
**B**	-6111.19963007	-6110.814515	-6110.913437	112.1	115.1	108.8	148.2	–
**TS1**	-6111.25561000	-6110.867844	-6110.968931	-34.8	-33.8	-33.1	0.6	-643
**C**	-6234.00028178	-6233.514966	-6233.620189	-57.1	-52.8	-51.2	0.6	–
**TS2**	-6233.97538000	-6233.492556	-6233.596434	8.3	13.6	8.6	64.0	-489
**D**	-6233.99477646	-6233.512050	-6233.616568	-42.6	-42.6	-47.9	5.8	–
**TS3**	-6233.99398348	-6233.51286	-6233.615158	-40.5	-33.9	-43.4	16.2	-158
**E**	-6234.05584972	-6233.570769	-6233.675616	-203.0	-197.6	-196.7	-143.9	–
**TS4**	-6234.03155525	-6233.551344	-6233.653734	-139.2	-131.8	-143.6	-84.3	-1387
**F**	-6234.05292759	-6233.566526	-6233.669948	-195.3	-195.3	-190.9	-134.3	–
**TS5**	-6234.04842577	-6233.566837	-6233.670109	-183.5	-175.6	-183.9	-126.9	-974
**G**	-6234.07671091	-6233.590582	-6233.694711	-257.7	-251.3	-247.6	-192.9	–

Table S5. Electronic energy, enthalpy, and Gibbs free energy for all the intermediates and TS structures in the **addrear2** mechanisms reported in Figure 1a related to the formation of phenol through electrophilic addition from a benzene molecule on a tri-iron oxo-centered metal node, as obtained at the UM06-L/def2-TZVP level on the pentadectet spin surface. These energies are reported as absolute values (E, H^0, G^0, in hartree) or referenced to the energies of the separated reagents (**A**, N_2O, and benzene) and corrected for BSSE (ΔE^c, ΔH^{0c}, ΔG^{0c}, in kJ mol^{-1}; for the electronic energy, the value not corrected for BSSE is also reported for comparison, ΔE). H^0 and G^0 have been calculated at 1 atm and 25 °C. The imaginary frequency $\tilde{\nu}_{imm}$ for the TS is also shown (in cm^{-1}).

**addrear2**	E	H^0	G^0	ΔE	ΔE^c	ΔH^{0c}	ΔG^{0c}	$\tilde{\nu}_{imm}$
**D’**	-6233.99511067	-6233.512167	-6233.616726	-43.5	-43.5	-48.2	5.4	–
**TS3’**	-6233.98567056	-6233.502713	-6233.606526	-18.7	-12.8	-17.4	38.2	-357
**E’**	-6234.01148935	-6233.526069	-6233.629605	-86.5	-81.2	-79.4	-23.1	–

Table S6. Electronic energy, enthalpy, and Gibbs free energy for all the intermediates and TS structures in the **phenolate** mechanisms reported in Figure 3 related to the formation of phenoxy species through the H-abstraction from phenol by a hydroxo species present on a tri-iron oxo-centered metal node, as obtained at the UM06-L/def2-TZVP level on the hexadectet spin surface. These energies are reported as absolute values (E, H^0, G^0, in hartree) or referenced to the energies of the separated reagents (**A-H** and phenol) and corrected for BSSE (ΔE^c, ΔH^{0c}, ΔG^{0c}, in kJ mol^{-1}; for the electronic energy, the value not corrected for BSSE is also reported for comparison, ΔE). H^0 and G^0 have been calculated at 1 atm and 298 K. The imaginary frequency $\tilde{\nu}_{imm}$ for each TS is also shown (in cm^{-1}).

phenolate	E	H^0	G^0	ΔE	ΔE^c	ΔH^{0c}	ΔG^{0c}	$\tilde{\nu}_{imm}$
**A (2S+1=15)**	-5926.50816949	-5926.135719	-5926.224777	–	–	–	–	–
**A-H**	-6002.35395001	-6001.965994	-6002.059546	–	–	–	–	–
**B**	-6309.9209693	-6309.419726	-6309.528056	-72.6	-66.5	-61.4	-7.5	–
**TS1**	-6309.90947159	-6309.413706	-6309.521434	-42.4	-35.5	-44.9	10.7	691*i*
**C**	-6309.91034436	-6309.410527	-6309.519530	-44.7	-39.4	-38.1	14.1	–
**TS2**	-6309.89716181	-6309.397380	-6309.503276	-10.0	-4.3	-3.1	57.3	81*i*
**D**	-6309.92453264	-6309.423114	-6309.533111	-81.9	-77.1	-71.6	-22.0	–
**D'**	-6309.93874799	-6309.436767	-6309.545141	-119.2	-112.8	-105.8	-52.0	–

S6. Additional Energetic Parameters

In Table S7, the absolute electronic energy, enthalpy, and free Gibbs energy for additional molecules present in the benzene to phenol reaction and not considered in the previous tables are reported.

Table S7. Electronic energy (E), enthalpy (H^0), and free Gibbs energy (G^0) obtained at the M06-L level for relevant molecules used in this study not reported in the other tables. All energies are reported in hartree. H^0 and G^0 have been calculated at 1 atm and 25 °C. All the energies are in hartree.

	$2S + 1$	E	H^0	G^0
C_6H_6	1	-232.2944000300	-232.188359	-232.221136
C_6H_5OH	1	-307.5393859730	-307.428012	-307.463352
N_2O	1	-184.7341688530	-184.719113	-184.743989
N_2	1	-109.5581970470	-109.549370	-109.571106

S7. Mass Spectrometric Data for Evaluation of Kinetic Isotope Effects

Mass spectra of phenol-h6 and deuterated phenol-d6 in water show that no fragmentation of these species occurs in solution (Figure S6). The data in Figure S7 show mass spectra of the product acquired using protium-form $^{13}C_6H_6$ and deuterated-$^{12}C_6D_6$ in independent experiments with varying O_2 pressures. These data are presented in tabular form in the manuscript as Table S8.

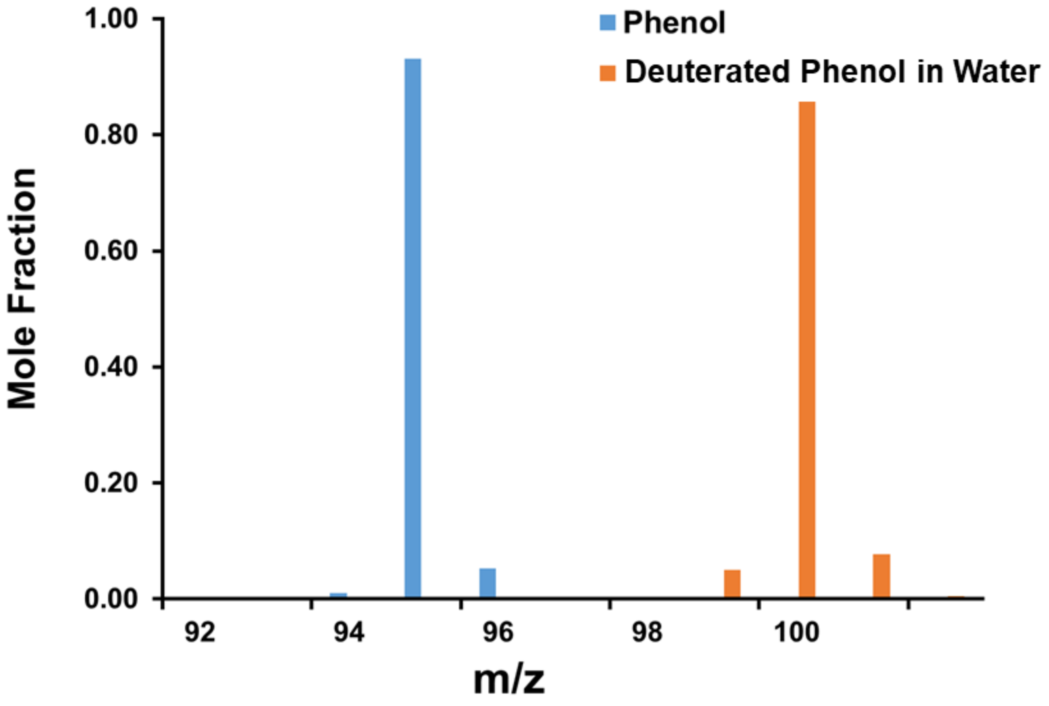

Figure S6. Mass spectra of phenol-h6 and deuterated phenol-d6 collected in H_2O, showing no fragmentation during MS analysis.

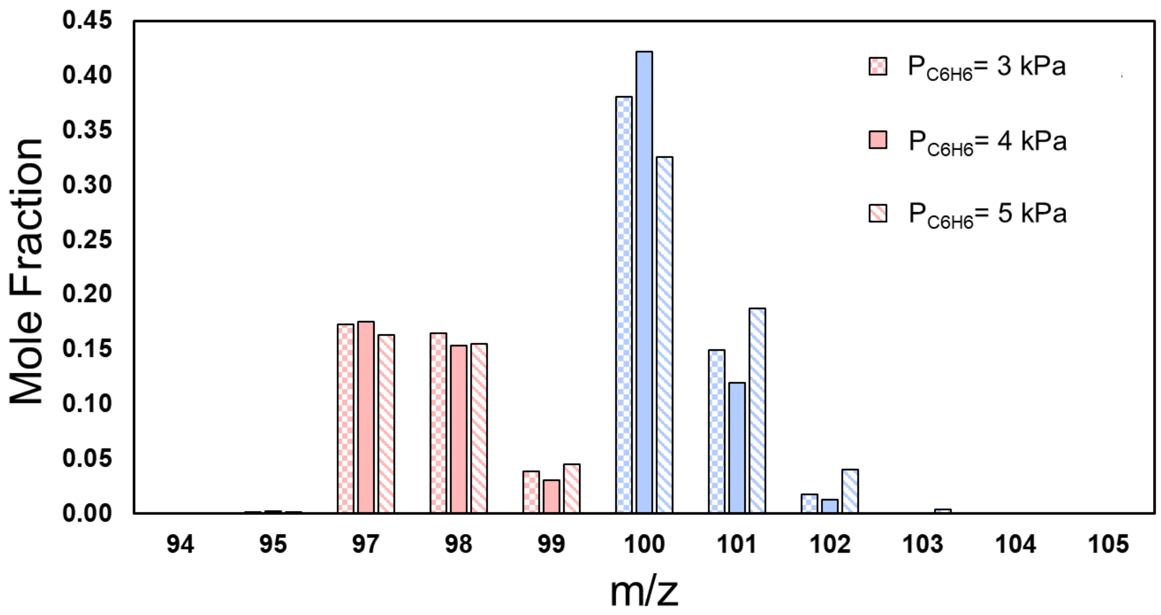

Figure S7. Mass spectra of the products in H_2O from experiments carried out with a mixture of protonated- and deuterated benzene ($^{13}C_6H_6$) and ($^{12}C_6D_6$) after exposure to 90 kPa N_2O +3-5 kPa benzene at 398 K for 2 h, followed by washing with H_2O ex-situ.

Table S8. Mole fractions of different isotopologues estimated by MS analysis of product extracted ex-situ in H_2O after exposure 90 kPa N_2O +3-5 kPa (Benzene) at 398 K for 2 h.

m/z	$\boldsymbol{P_{C6H6}}$ **= 3 kPa**	$\boldsymbol{P_{C6H6}}$ **=4 kPa**	$\boldsymbol{P_{C6H6}}$ **= 5 kPa**
94	0.00	0.00	0.00
95	0.00	0.00	0.00
96	0.07	0.08	0.08
97	0.17	0.18	0.16
98	0.17	0.15	0.15
99	0.04	0.03	0.05
100	0.38	0.42	0.33
101	0.15	0.12	0.19
102	0.02	0.01	0.04
103	0.00	0.00	0.00
104	0.00	0.00	0.00
105	0.00	0.00	0.00
$r_D X_D{}^{a,b}$	0.45	0.45	0.44
$r_H X_H{}^{a,b}$	0.55	0.55	0.56
**KIE**	**0.83**	**0.84**	**0.85**

[a] r_H and r_D refers to the rate of the protonated (100 < *MW* < 105) or deuterated benzene (94 < *MW* < 99), respectively.

[b] X_H and X_D refer to the conversion of the protonated (100 < *MW* < 105) or deuterated benzene (94 < *MW* < 99), respectively.

S8. Reactor Unit set-up

A schematic representation of the reaction unit which can operate both as a flow reactor and as a recirculating batch reactor is shown in Figure S8 below. This external recycle loop allows the system to operate as a batch reactor so far as low single pass conversion is achieved in each pass through over the reactor.

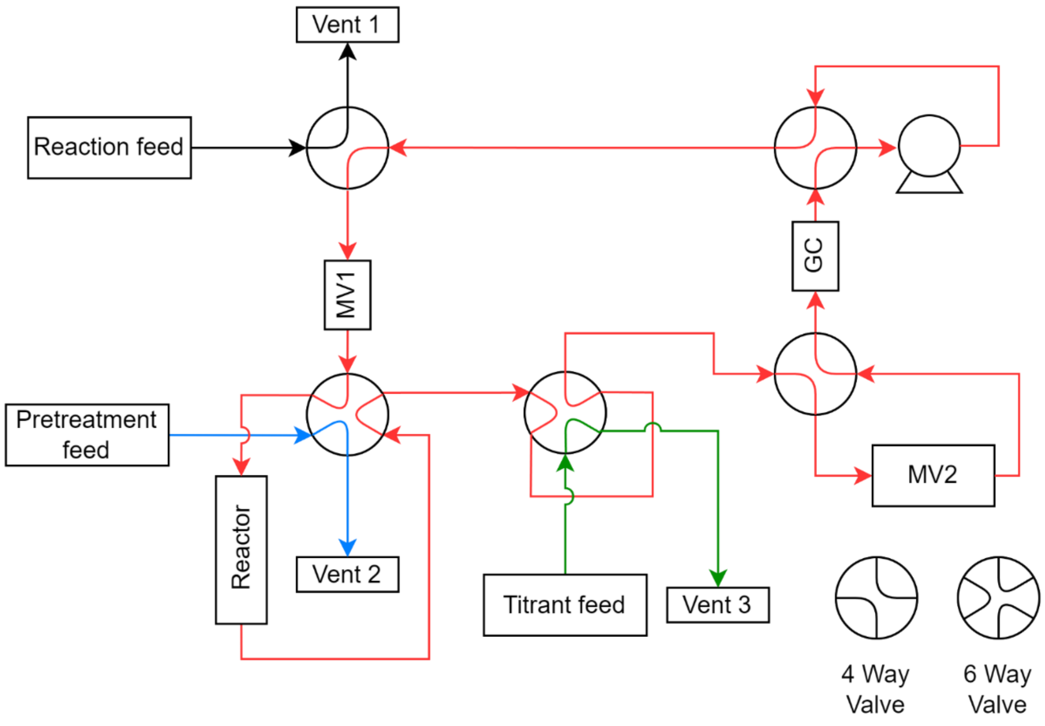

Figure S8. Scheme of the gas phase recirculating batch reactor used in the experiments.

References

1. Simons, M. C.; Vitillo, J. G.; Babucci, M.; Hoffman, A. S.; Boubnov, A.; Beauvais, M. L.; Chen, Z.; Cramer, C. J.; Chapman, K. W.; Bare, S. R.; Gates, B. C.; Lu, C. C.; Gagliardi, L.; Bhan, A., Structure, Dynamics, and Reactivity for Light Alkane Oxidation of Fe(II) Sites Situated in the Nodes of a Metal–Organic Framework. *J. Am. Chem. Soc.* **2019,** *141* (45), 18142-18151.
2. Gaggioli, C. A.; Stoneburner, S. J.; Cramer, C. J.; Gagliardi, L., Beyond Density Functional Theory: The Multiconfigurational Approach To Model Heterogeneous Catalysis. *ACS Catal.* **2019,** *9* (9), 8481-8502.
3. Vitillo, J. G.; Bhan, A.; Cramer, C. J.; Lu, C. C.; Gagliardi, L., Quantum Chemical Characterization of Structural Single Fe(II) Sites in MIL-Type Metal–Organic Frameworks for the Oxidation of Methane to Methanol and Ethane to Ethanol. *ACS Catal.* **2019,** *9* (4), 2870-2879.
4. Vitillo, J. G.; Lu, C. C.; Cramer, C. J.; Bhan, A.; Gagliardi, L., Influence of First and Second Coordination Environment on Structural Fe(II) Sites in MIL-101 for C–H Bond Activation in Methane. *ACS Catal.* **2021,** *11* (2), 579-589.
5. de Visser, S. P.; Shaik, S., A Proton-Shuttle Mechanism Mediated by the Porphyrin in Benzene Hydroxylation by Cytochrome P450 Enzymes. *J. Am. Chem. Soc.* **2003,** *125* (24), 7413-7424.
6. de Visser, S. P.; Oh, K.; Han, A.-R.; Nam, W., Combined Experimental and Theoretical Study on Aromatic Hydroxylation by Mononuclear Nonheme Iron(IV)−Oxo Complexes. *Inorg. Chem.* **2007,** *46* (11), 4632-4641.
7. Snyder, B. E. R.; Bols, M. L.; Rhoda, H. M.; Vanelderen, P.; Böttger, L. H.; Braun, A.; Yan, J. J.; Hadt, R. G.; Babicz, J. T.; Hu, M. Y.; Zhao, J.; Alp, E. E.; Hedman, B.; Hodgson, K. O.; Schoonheydt, R. A.; Sels, B. F.; Solomon, E. I., Mechanism of selective benzene hydroxylation catalyzed by iron-containing zeolites. *Proc. Natl. Acad. Sci. U. S. A.* **2018,** *115* (48), 12124-12129.
